# NU9056, a KAT 5 Inhibitor, Treatment Alleviates Brain Dysfunction by Inhibiting NLRP3 Inflammasome Activation, Affecting Gut Microbiota, and Derived Metabolites in LPS-Treated Mice

**DOI:** 10.3389/fnut.2021.701760

**Published:** 2021-07-13

**Authors:** Lu Chen, Wenxiang Qing, Zexiong Yi, Guoxin Lin, Qianyi Peng, Fan Zhou

**Affiliations:** ^1^Department of Anesthesiology, The Third Xiangya Hospital, Central South University, Changsha, China; ^2^Medical College of Xiangya, Central South University, Changsha, China; ^3^Department of Critical Care Medicine, Xiangya Hospital, Central South University, Changsha, China; ^4^Department of Anesthesiology, The Second Xiangya Hospital, Central South University, Changsha, China

**Keywords:** NU9056, gut microbiota, NLRP3 inflammasome, derivative metabolites, sepsis-associated encephalopathy

## Abstract

**Background:** The pathogenesis of sepsis-associated encephalopathy (SAE) is complicated, while the efficacy of current treatment technologies is poor. Therefore, the discovery of related targets and the development of new drugs are essential.

**Methods:** A mouse model of SAE was constructed by intraperitoneal injection of lipopolysaccharide (LPS). LPS treatment of microglia was used to build an *in vitro* model of inflammation. Nine-day survival rates, behavioral testing, transmission electron microscopy (TEM), immunohistochemical (IHC), immunofluorescence (IF), and ELISA were performed. The expression levels of Occludin, Claudin 5, NLRP3, caspase-1, and ASC genes and proteins were detected by RT-qPCR or Western blot. Caspase-1 P10 (Casp-1 P10) protein expression was detected. 16S rDNA sequencing and gas chromatography-mass spectrometer (GC-MS) were used to analyze the gut microbiota and metabolism. Flow cytometric experiment and Cell Counting Kit-8 (CCK8) assay were performed.

**Results:** NU9056 improved the survival rate of mice and alleviated LPS-induced cognitive impairment, anxiety, and depression *in vivo*. The tight junctions were thickened via NU9056 treatment. Further, the mRNAs and proteins expression levels of Occludin and Claudin 5 were up-regulated by NU9056. NU9056 increased the expression level of DCX. The expression levels of Iba-1, NLRP3, IL-1β, ASC, and Casp-1 P10 were down-regulated by NU9056. The composition of the gut microbiota changed. Kyoto Encyclopedia of Genes and Genomes data predicted that the effects of NU9056 might be related to apoptosis and tight junction pathways. NU9056 up-regulated the concentration of acetate, propionate, and butyrate. NU9056 significantly reduced LPS-induced apoptosis of microglia, the average fluorescence intensity of ROS, and the release of IL-1β and IL-18, while improving cell viability *in vitro*.

**Conclusions:** NU9056 might effectively alleviate LPS-induced cognitive impairment and emotional disorder in experimental mice by inhibiting the NLRP3 inflammasome. The therapeutic effects may be related to gut microbiota and derived metabolites. NU9056 might be a potential drug of SAE prevention.

## Introduction

Sepsis is one of the leading causes of death in intensive care units (ICU) worldwide and often causes neurological disorders, such as sepsis-associated encephalopathy (SAE). SAE is characterized by pro-inflammatory and anti-inflammatory imbalance, multiple organ dysfunction, severe nervous system disorder, and cognitive and mental dysfunction (1). Although progress has been made in drug therapy and surgical treatment, and age-standardized morbidity and mortality rates have been declining, patients with SAE are still severely. They have a high mortality rate (2). Knowledge of the pathogenesis of sepsis is incomplete, and the discovery of SAE-related targets and the development of corresponding drugs remain crucial goals.

Blood-brain barrier (BBB) damage is closely associated with neuronal damage in SAE (3). Destruction of the BBB is also accompanied by the activation of the NLR family pyrin domain containing 3 (NLRP3, previously known as NACHT, LRR, and PYD domain-containing protein 3) inflammasome (4). The NLRP3 inflammasome is mainly composed of an intracellular sensor, NLRP3, an adaptor ASC and an effector caspase-1 (5). The NLRP3 inflammasome plays a vital role in neuro-inflammatory diseases, like Alzheimer's and Parkinson's disease (6). Related inhibitors, such as the NLRP3 inhibitor MCC950 and the caspase-1 inhibitor VX765, can significantly reduce the neuro-inflammatory damage caused by SAE (7, 8).

Intestinal flora and metabolic disorders are also major factors in the deterioration of SAE. Maintaining metabolic homeostasis contributes to the effective treatment of SAE (9, 10). Probiotics, such as *Clostridium butyricum*, may improve the cognitive impairment of SAE mice by regulating the gut microbiota (11). Related reports have indicated that the gut microbiota of NLRP3-deficient mice can improve depression by regulating astrocytes (12). The NLRP3 inflammasome is also a sensor of metabolic stress (13). Thus, inhibition of the NLRP3 inflammasome may have the potential for SAE treatment by regulating gut microbiota and metabolism.

The KAT5 (also known as Tip60), H4K16 histone acetyltransferase, is present in hypoxia-reoxygenation macrophages. The overexpression of KAT5 and myocardin-related transcription factor A (MRTF-A) synergistically activate the pro-inflammatory factor-induced nitric oxide synthase (iNOS) (14). NLRP3 self-aggregation and complete inflammasome activation require acetylation (15).

NU9056 is a specific inhibitor of KAT5 (16) related to the inhibition of NLRP3 inflammasome (15). However, relatively little is known regarding the underlying mechanism. In the present study, we investigated whether NU9056 has a significant therapeutic effect on SAE *in vivo* and *in vitro* and the main possible reasons for these effects.

## Materials and Methods

### Animal Model

Ninety-five, 12-week-old C57BL/6J mice weighing 25–30 g were randomly divided into a control group, a lipopolysaccharide (LPS) group, and an NU9056 (LPS+NU9056, L. Nu) group. This study was approved by the Institutional Animal Care and Use Committee of the Third Xiangya Hospital, Central South University (No: LLSC (LA) 2018-035). The mice were purchased from Hunan Slack Jingda Experimental Animal Co., Ltd. Laboratory. The animals were adaptively fed for 7 days from the date of purchase. They were reared at room temperature (25 ± 2°C). The relative humidity was ~55%. Alternating 12 h cycles of light and dark were used. Mice had free access to food and water. LPS-treated mice received an intraperitoneal injection of LPS (10 mg/kg, cat# L2880, Sigma). Control mice were injected with the same amount of normal saline. NU9056 (5 mg/kg, cat#4903, TOCRIS) was intraperitoneally injected twice, 30 min before and 24 h after LPS injection. Sixteen hours after intraperitoneal injection of LPS, blood was collected transcardially. The supernatant was separated and collected. The experiment was performed immediately. Finally, the remaining samples were stored at −80°C. According to the process shown in Supplementary Figure 1, the brains and feces from the end of the colon were collected. A portion of the samples were fixed and tested, and the remainder were stored at −80°C. Another 43 mice were randomly divided into three groups with the same grouping and treatment methods as well as before. The animal survival rate and behavioral experiments were carried out. The mice were euthanized by intraperitoneal injection of sodium pentobarbital 150 mg/kg.

### Cell Culture and Treatment

BV2 cells (cat# ZQ0397, Shanghai Zhong Qiao Xin Zhou Biotechnology Co., Ltd.) were cultured in DMEM (cat# C11995500BT, Gibco), supplemented with 10% fetal bovine serum (cat# 10099141, Gibco), 100 IU/mL penicillin, and 100 μg/mL streptomycin sulfate (cat# C0222, Beyotime Biotechnology). The cells were placed in a cell culture incubator in an atmosphere of 5% CO_2_ at 37°C. Cells were divided into the control group, LPS, and L. Nu groups. The cells in the control group were treated with the same volume of solvent as LPS in the culture medium. The cells in the LPS group were pre-treated with a volume of solvent equivalent to the NU9056 volume for 30 min and followed by the addition of 1 μg/mL LPS and culture of the cells for 24 h. The cells of the L. Nu group were pre-treated with NU9056 (10 μM) for 30 min prior to the same treatment with LPS.

### Survival of Animals and Behavioral Testing

The mice were observed daily for their survival. Animal behavior experiments included an open field test (OFT), novel object recognition (NOR), elevated plus maze (EPM), and mouse tail suspension test (TST). The tests were performed as described previously (7) and are briefly described below.

#### OFT

The open-field box was 40 ×40 ×40 cm. The total distance traveled in five min was analyzed using Smart Junior software (version 3.0; Panlab, Spain).

#### NOR

Each mouse was placed in a square space of 40 ×40 ×40 cm and underwent familiarization and discrimination. Each mouse could explore 10 min in the field in the familiarization phase with two identical objects (A1 and A2) located opposite and equidistant positions. Twenty-four hours later, each mouse was returned to the open field where one of the familiar objects (A2) was replaced by a novel object (A3). In the discrimination phase, each mouse could explore objects for 10 min, and the time of exploring each object was recorded. Preference indexes of training and test were analyzed.

#### EPM

The plus-maze height of 50 cm included a central square, two open arms, and two closed arms. The two closed arms were 30 cm in length and 5 cm in width and enclosed by walls with a height of 15 cm. Open arms had no walls. Mice were placed in the central square facing one of the open arms and allowed to explore individually for 5 min. The total time spent in open arms was calculated using the Smart Junior software (version 3.0; Panlab, Spain).

#### TST

The mice were fixed with the tip of their tail on a horizontal scaffold at the height of 50 cm with the head down. Next, the duration of immobility was recorded for 6 min by the Smart Junior software (version 3.0; Panlab, Spain).

### Transmission Electron Microscopy (TEM)

As previously mentioned (17, 18), the samples were fixed in 2.5% glutaraldehyde for 2 h. The samples were washed three times with phosphate-buffered saline (pH 7.2–7.4). The samples were exposed to 1% osmium tetroxide for 1.5 h and then dehydrated. The samples were infiltrated using Poly/Bed 812 resin. TEM was performed using a model H-7700 transmission electron microscope (Hitachi).

### Quantitative Reverse Transcription-Polymerase Chain Reaction (RT-qPCR)

RNA was extracted from tissues and cells using TRIzol (Invitrogen) according to the manufacturer's instructions. Next, the extracted RNA was reverse-transcribed into cDNA. The sequences of the target genes were searched using NCBI, and the primers were designed using Primer 5 software (Premier). The primer sequences for each gene were shown in Table 1. The relative expression of each target gene was calculated using the 2^−ΔΔCt^ method with β-actin as the internal reference.

**Table 1:** All primer sequences were used in the study.

**Gene**	**Sequences (5^′^-3^′^)**
Occludin	F:GTTAAGGCACGGGTAGCACT
	R:TACTTCTGTGACACCGGCAC
Claudin	F: GTTAAGGCACGGGTAGCACT
	R: TACTTCTGTGACACCGGCAC
NLRP3	F: CCTCTTTGGCCTTGTAAACCAG
	R: TGGCTTTCACTTCAATCCACT
ASC	F: CAGAGTACAGCCAGAACAGGACACT
	R: AAGCATCCAGCACTCCGTCCAC
Caspase-1	F: ACAAGGCACGGGACCTATG
	R: TCCCAGTCAGTCCTGGAAATG
β-actin	F: ACATCCGTAAAGACCTCTATGCC
	R: TACTCCTGCTTGCTGATCCAC

### Western Blot

Total proteins in each group of tissues were extracted and denatured. After sodium dodecyl sulfate-polyacrylamide gel electrophoresis, the proteins were transferred to nitrocellulose membranes. The membranes were incubated overnight at 4°C with primary antibodies to Occludin (1:2,000, cat# 27260-1-AP, Proteintech), Claudin5 (1:2,000, cat# ab131259, Abcam), NLRP3 (1:1,000, cat# ab263899, Abcam), ASC (1:1,000, cat# AG-25B-0006-C100, Adipogen), Casp-1(1:1,000, cat#24232, Cell Signaling Technology), and β-actin (1:5,000, cat# 60008-1-Ig, Proteintech). The membranes were then exposed to secondary antibody horseradish peroxidase (HRP)-conjugated goat anti-mouse IgG (1:5,000, cat# SA00001-1, Proteintech) or HRP goat anti-rabbit IgG (1:6,000, cat# SA00001-2, Proteintech) was incubated for 90 min at room temperature. β-Actin was used as an internal control. After ECL color exposure, the protein bands were analyzed using an Odyssey infrared imaging system (Li cor Biosciences).

### Immunohistochemistry (IHC)

Tissue sections were first deparaffinized and heat-repaired for antigen retrieval and for other routine treatments. The sections were incubated in 3% H_2_O_2_ for 25 min to remove endogenous peroxidase activity and blocked in 3% BSA for 30 min at room temperature. The primary antibody doublecortin (DCX, cat# 4604S, Cell Signaling Technology) diluted 1:500 was added and incubated overnight at 4°C. The sections were rinsed in phosphate-buffered saline and incubated with goat anti-rabbit secondary antibody (100 μL; cat# PV-9000, ZSGB-BIO) at room temperature for 50 min. Subsequently, the avidin-biotin-peroxidase complex (ABC Elite Kit, Vector Laboratories) was added at room temperature. Positive expression was visualized using enhanced 3,3′ diaminobenzidine.

### Immunofluorescence (IF)

Tissue sections were first deparaffinized and heat-repaired for antigen retrieval and for other routine treatments. The cell slides were fixed and permeabilized by routine processing. The primary antibodies Iba1 (1:100; cat# 10904-1-AP, Proteintech) and NLRP3 (2 μg/ml; cat# PA5-79740, ThermoFisher) were incubated overnight at 4°C. The next day, goat anti-rabbit IgG (1:200; cat# SA00013-2, Proteintech) was incubated at 37°C for 90 min. In addition, a 4′,6-diamidino-2-phenylindole (DAPI) working solution was used to stain the nucleus for 10 min. Finally, buffered glycerol was used to mount the slides, and the samples were stored in the dark and observed under a fluorescence microscope.

### ELISA

Serum and cellular supernatants were collected. ELISA detection kit for interleukin (IL)-18 (cat# CSB-E04609m) was purchased from Cusabio Biotech Co., Ltd. ELISA detection kits, including IL-1β (cat#88-7013-77) and IL-6 (cat# 88-7064-77), were purchased from eBioscience. The concentrations of IL-1β, IL-6, and IL-18 were determined according to the manufacturer's instructions. A microplate reader (MB-530, Shenzhen Huisong Technology Development Co., Ltd.) was used to measure the optical density (OD) value of each well at 450 nm within 5 min after the termination of the reaction. The sample concentration was determined using a regression equation of the standard curve.

### 16S rDNA Sequencing Analysis

DNA was extracted following the stool genomic DNA kit instructions (cat# DP328-02, TIANGEN). The concentration of DNA using the dsDNA HS Assay Kit (cat# 12640ES76, Shanghai Yisheng Biotechnology Co., Ltd.) was measured. 16S rDNA sequencing was performed using a NovaSeq PE250 device (Illumina). Raw data were obtained and subjected to unlinking, filtering, deduplication, base correction, and removing the chimera sequence to obtain a valid sequence (clean data) for subsequent analysis. Sequence data were assessed using Qiime 2 (Qiime2-2020.2) and R software (4.0.2). Based on the Kyoto Encyclopedia of Genes and Genomes (KEGG) gene function spectrum data, the conversion calculation of the total metabolic function of the flora was performed and analyzed via KEGG differentiation pathway analysis.

### Fecal Short-Chain Fatty Acid (SCFA) Detection

The concentration of SCFA consisting of acetate, propionic, isobutyric, butyric, isovalerate, and valerate were measured via gas chromatography-mass spectrometry (GC-MS) using a model 5977 B apparatus (Agilent). An appropriate amount of feces was added to 300 μL of normal saline containing 37.3 μg/mL d7 isobutyric acid and magnetic beads and homogenized at 60 Hz for 60 s. After centrifugation, acidification, extraction, and other treatments, the samples were analyzed using GC-MS with a DB-WAX capillary column (30 m ×0.250 mm ×0.25 μm) and 99.999% helium as the chromatographic carrier gas at a flow rate of 1 mL/min. The temperature of the injection port and auxiliary heater was 250 and 260°C, respectively. The oven temperature was programmed to start at 50°C and was increased at different rates. Finally, the temperature was increased to 240°C at a rate of 15°C/min and maintained for 5 min. The scanning range was 33–300 Da. The concentration of SCFA was quantified based on the peak area of the total ion current.

### Flow Cytometric Experiment

The cells were collected after the abovementioned treatment. An Annexin V-FITC apoptosis detection kit (cat# KGA108, Nanjing KGI) was used to treat the cells according to the manufacturer's instructions. Apoptosis rates were analyzed using a flow cytometer (A00-1-1102, Beckman). The cells were treated with 10 μM of 2′,7′-dichlorofluorescein diacetate (DCFH-DA; cat# S0033S, Beyotime Biotechnology) and incubated at 37°C for 20 min. The fluorescence intensity of reactive oxygen species (ROS) was measured using flow cytometry.

### Cell Counting Kit-8 (CCK8) Assay

Cells in the logarithmic growth phase were digested and counted. They were seeded in a 96-well plate at a density of 5 ×10^3^ cells/well using 100 μL per well, with five replicate wells in each group. After 24 h, the cells were processed according to the above groups. The assay was performed according to the manufacturer's protocol. OD_450nm_ was measured on a microplate reader (Bio-Tek). The average value was calculated, and the survival rate curve was plotted.

### Statistical Analyzes

Statistical analyzes were performed using GraphPad Prism 8 software (GraphPad Software). Unpaired *t*-tests were used to determine the statistical significance between the two groups. Three or more groups were determined using a one-way analysis of variance. Data are expressed as mean ± standard deviation (SD). Significance was indicated by a *P* < 0.05.

## Results

### NU9056 Improved Survival Rate and Relieved Cognitive Dysfunction and Emotional Disorder in LPS-Induced Mice

**Figure 1:**
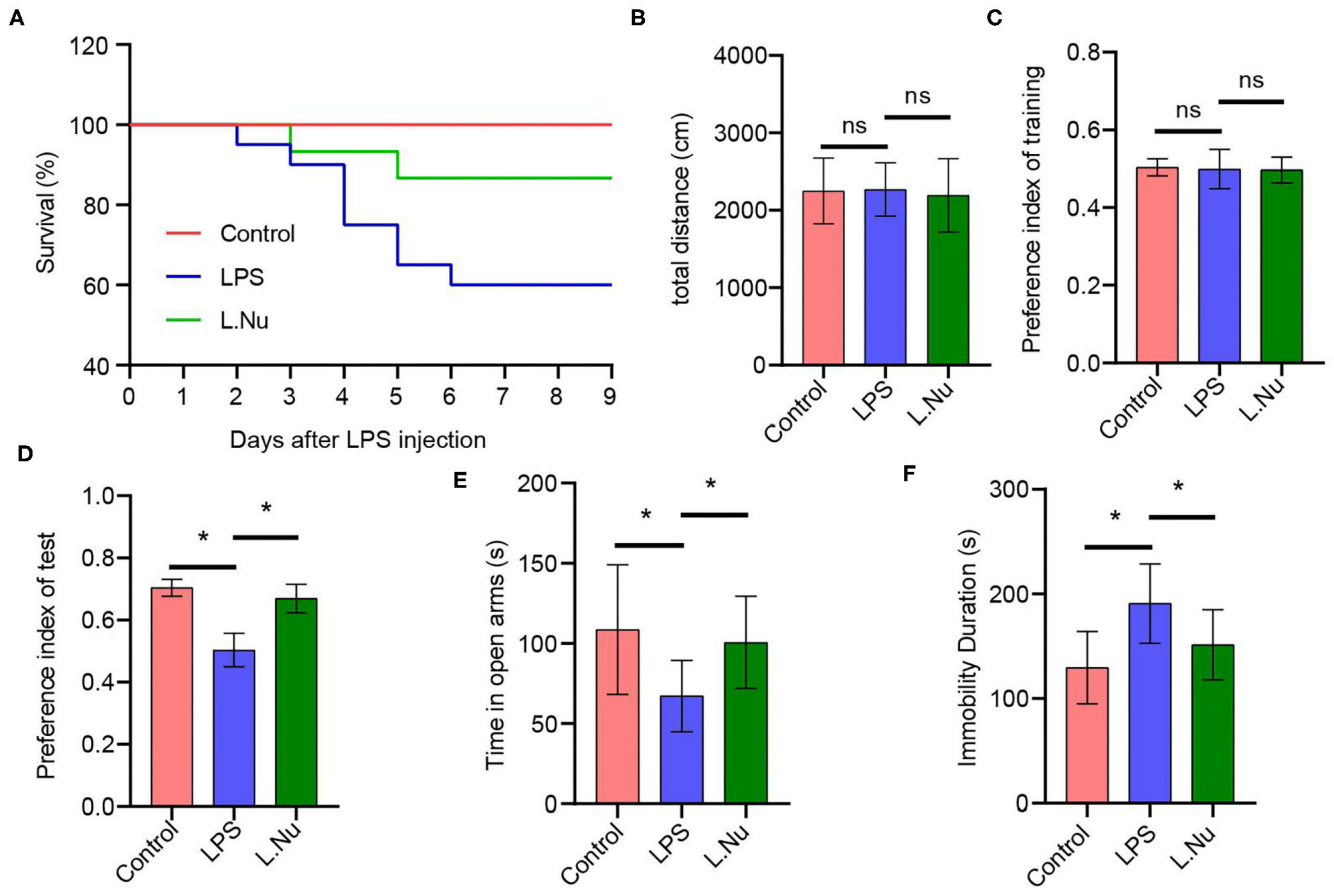
NU9056 improved survival rate and relieved cognitive and emotional dysfunction in LPS-induced mice. **(A)** The 9-day survival rate of mice was determined. Twelve of twenty mice of the LPS group survived, 13 of 15 mice of the L. Nu group survived. **(B)** The total distance moved by each group of mice was detected in the OFT experiment. **(C,D)** The preference index of training and test were recorded in the NOR experiment. **(E)** The time in open arms was detected in the EPM assay. **(F)** The duration of immobility of the mice was analyzed using the TST experiment. *P < 0.05; ns, not significant; *n* = 8–20 mice/group.

In order to identify the effects of NU9056 on LPS-induced mice, a survival analysis was conducted. The results found that the survival percent in all of the animals in the control group was 100% during the 9 days in the study, suggesting they were normal. In contrast, the animals in the LPS group began to die the next day and continued to die on days 2–6, with an eventual survival rate of 60%. In the L. Nu group, the survival curve was relatively flat compared to the LPS group, and the eventual survival rate was 87% (Figure 1), suggesting that NU9056 has noticeable therapeutic effects in the LPS-induced mice. To verify the impact of NU9056 on cognitive and emotional dysfunction, OFT, NOR, EPM, and TST behavioral experiments were performed according to the experimental shown in Supplementary Figure 1. The OFT experiment results showed that there was no significant difference in the total distance moved by the mice in each group 5 days after LPS injection (Figure 1B). The results of the NOR experiment indicated that the preference index of training in the mice among the groups was not different for the left and right objects. In the testing phase, NU9056 increased the exploration index of the novel object induced by LPS in mice (Figures 1C,D). In the EPM experiment, the time in the open arms of the mice in the LPS group was significantly reduced compared with that in the control group, while the time for the mice in the L. Nu group increased significantly compared with that in the LPS group (Figure 1E). The TST results showed that the immobility duration of the L. Nu group markedly lower than that of the LPS group (Figure 1F). The collective results indicated that NU9056 reversed cognitive dysfunction and emotional disorder of mice in the LPS group.

### NU9056 Might Inhibit Microglia Activation and Protect From BBB Damage by Downregulating the NLRP3 Inflammatory Pathway in the Mice Treated With LPS

**Figure 2:**
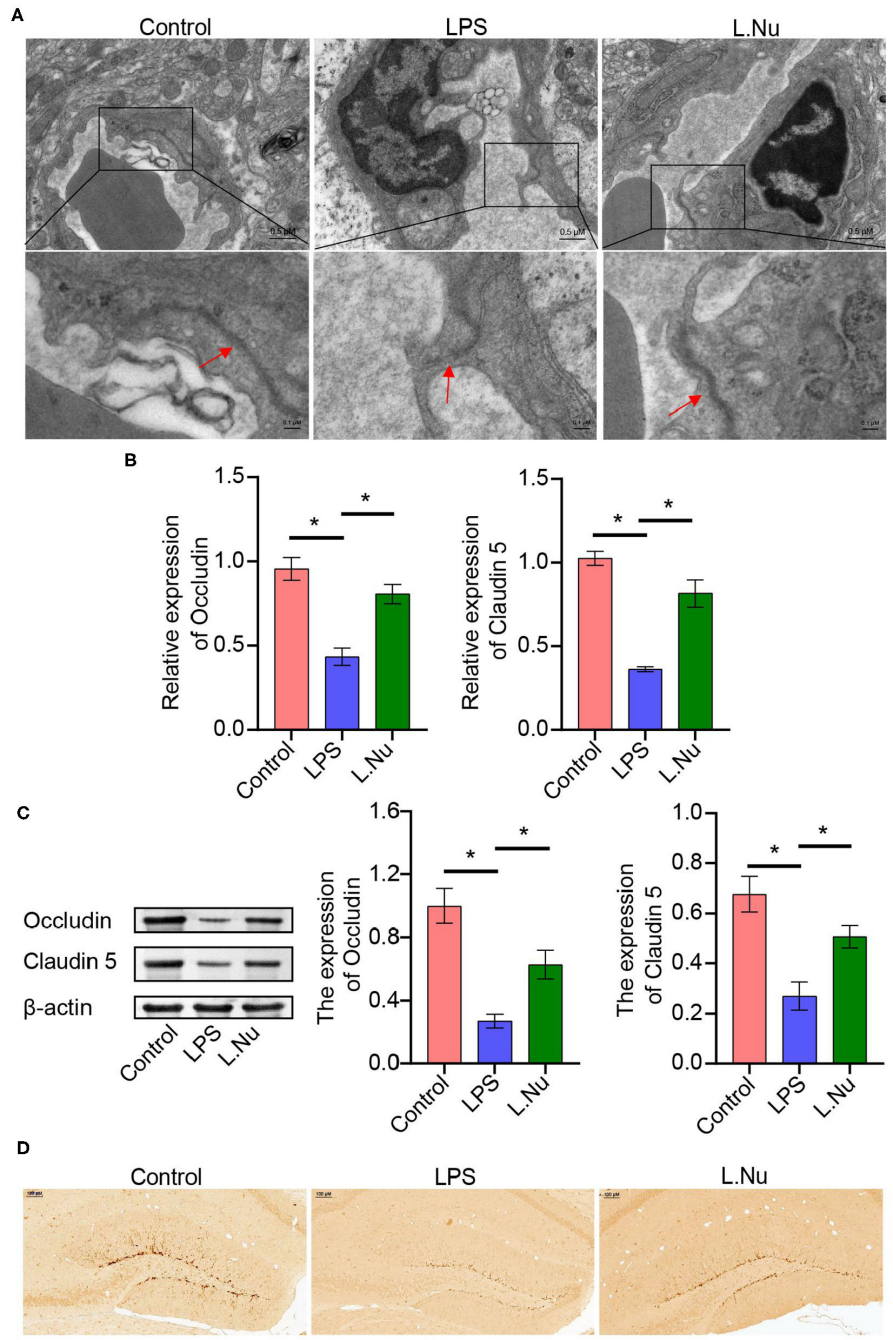
NU9056 protected BBB, and newborn neurons function from damage *in vivo*. **(A)** The morphological changes of the tight junctions of the hippocampus in the mice were observed using TEM. The arrow points to a tight junctions structure. **(B,C)** The expression levels of Occludin and Claudin 5 genes and proteins in the mouse hippocampus were detected using RT-qPCR and Western blot. **(D)** The DCX expression level of newborn neurons in the brain tissue of mice was observed using IHC. *P < 0.05.

TEM revealed that the tight junctions of the LPS group showed local thinning, indicating that the tight junctions and BBB were damaged. Compared with the mice in the LPS group, the tight junctions of the mice in the NU9056 group became thicker, and the structure tended to be normal, indicating that the BBB function was protected (Figure 2A). RT-qPCR and Western blot were used to explore the BBB function and molecular pathways related to NU9056. The expression levels of genes and proteins, including Occludin and Claudin 5 in the L. Nu group, were significantly higher than those in the LPS group (Figures 2B,C). To further verify the effects of NU9056 in LPS-induced mice on newborn brain neurons, IHC was performed. The results indicated that the L. Nu group reversed the LPS-induced decrease in the expression level of DCX, suggesting that NU9056 has a potential therapeutic effect in mice with SAE (Figure 2D). Altogether, NU9056 protected BBB and newborn neurons' function from damage *in vivo*.

**Figure 3:**
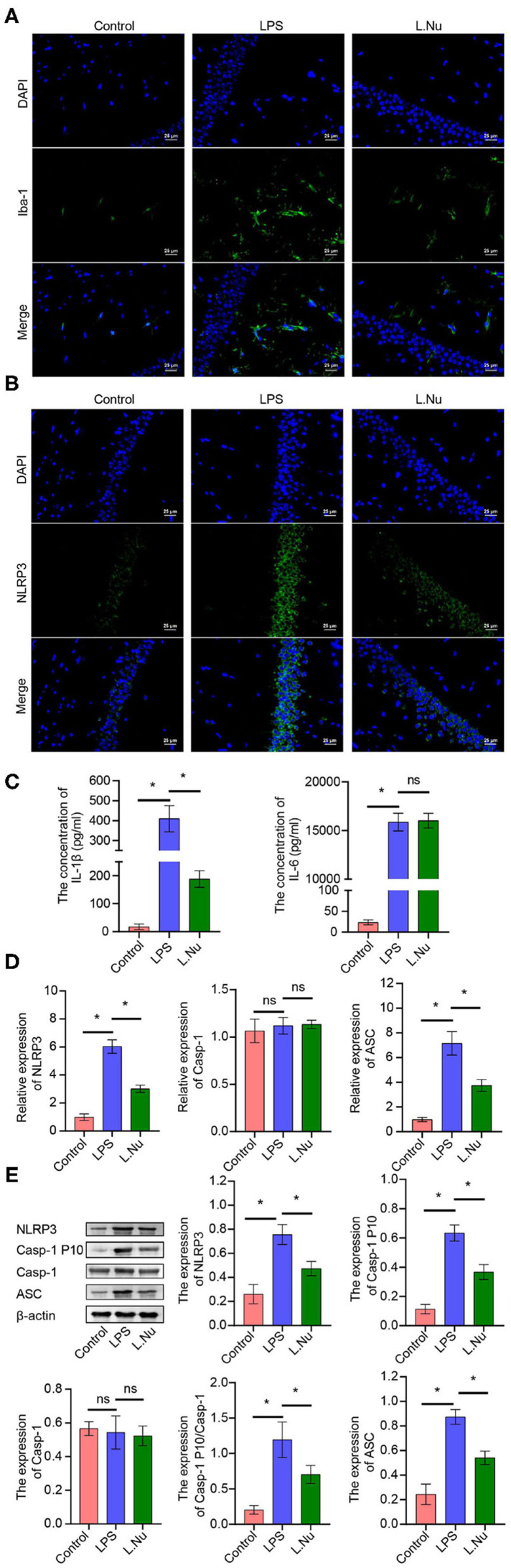
NU9056 might inhibit microglia activation and inflammation by inhibiting the NLRP3 inflammatory pathway *in vivo*. **(A,B)** The expression level of Iba-1 and NLRP3 in hippocampal microglia of mice were detected using IF; **(C)** the concentrations of IL-1β and IL-6 in the serum of mice were tested using ELISA; **(D,E)** genes or proteins expression levels of NLRP3, ASC, Casp-1, and Casp-1 P10 in the hippocampus of mice were analyzed by RT-qPCR or Western blot. *P < 0.05; ns, not significant.

To validate whether NU9056 activated hippocampal microglia, *in vivo* IF was performed. Compared with the control group, NU9056 reversed the abnormal activation of Iba-1 and NLRP3 in the hippocampus of mice stimulated with LPS (Figures 3A,B). In contrast to the LPS group, the expression level of the serum inflammatory factor IL-1β in the L. Nu group was significantly reduced, while there was no significant difference in IL-6 levels (Figure 3C). Moreover, in the hippocampus of mice, related inflammation and pyrolysis pathway indicators, the expression levels of NLRP3 and ASC genes and proteins were abnormally activated, and that of Casp-1 splicing body P10 protein was significantly increased. However, NU9056 reversed the above process (Figures 3D,E). In addition, the results suggested that NU9056 might inhibit the abnormal activation of microglia and inflammation induced by LPS by down-regulating the NLRP3 inflammatory pathway.

### NU9056 Affected Fecal Microbiota in LPS-Induced Mice

**Figure 4:**
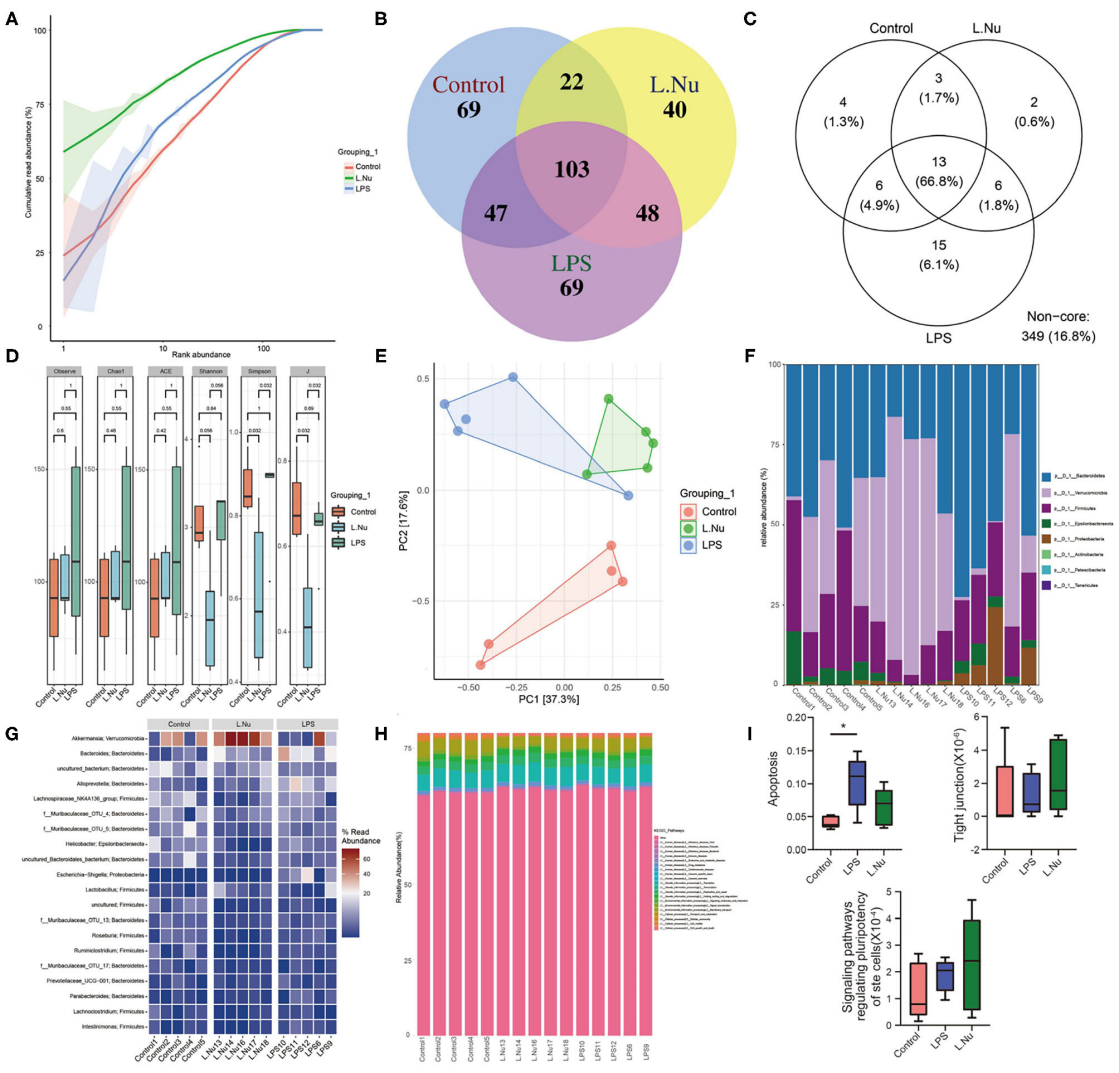
NU9056 affected the fecal microbiota in LPS-induced mice. **(A)** Rank-abundance graph. **(B)** Venn diagram of the number of common and unique OTUs in each group. **(C)** Venn diagram of the number of common and unique core species OTUs in each group. **(D)** The alpha diversity of gut microbiota in each group was analyzed using 16S rDNA sequencing, consisting of (1) Observed OTUs, (2) Chao1 index, (3) ACE index, (4) Shannon index, (5) Simpson's index, and (6) J index. **(E)** Principal component analysis of the similarity of samples among groups. **(F)** Variation in the relative abundance of samples in each group at the phylum level. **(G)** Variation in the relative abundance of samples in each group at the genus level. **(H)** KEGG pathways predictions of functions of gut microbiota at the class level. **(I)** KEGG pathways predictions of enrichment of each group in apoptosis, tight junction, and signaling pathways regulating the pluripotency of stem cells. *P < 0.05.

To further identify whether the effects of NU9056 in alleviating SAE were related to the gut microbiota, 16S rDNA sequencing was performed. The rank-abundance graph showed that as the sequencing depth increased, the read abundance gradually increased and finally tended to be flat, indicating that each group of samples' species richness and uniformity were eligible (Figure 4A). The operational taxonomic unit (OTU) species annotation Venn diagram indicated that in terms of overall species diversity, the species abundance of the L. Nu group decreased (Figure 4B). The results of an OTU core species annotation Venn diagram suggested that compared with the control group, the endemic species clusters of the LPS group were up-regulated, while that of the L. Nu group tended to be similar to the normal group (Figure 4C). We then analyzed the alpha diversity of the samples among the groups. Compared with the LPS group, the Shannon, Simpson's, and J indices of the L. NU group decreased significantly, indicating that biodiversity was reduced (Figure 4D). The beta diversity results of the samples demonstrated that the degree of dispersion between LPS groups was greater, whereas it was significantly reduced after NU9056 treatment (Figure 4E). Subsequently, we analyzed the changes in fecal microbial abundance at the phylum and genus levels. At the phylum level, compared with the LPS group, the relative abundance of the *Verrucomicrobia* phylum in the L. Nu group showed an upward trend (Figure 4F). At the genus level, the abundance of the *Akkermansia* genus in the phylum *Verrucomicrobia* in the L. Nu group was notably higher than that in the LPS group (Figure 4G). The above results suggested that the mitigation effects of NU9056 might be related to the diversity and structural changes in the gut microbiota.

To further distinguish the function of changes caused by changes in species abundance, KEGG pathways were used for functional predictions. The results of data analysis at the level of the Class predicted a changing trend of cellular processing pathway enrichment in the L. Nu group (Figure 4H). Compared to the control group, the apoptosis signaling pathway in the LPS group was significantly enriched. In addition, the tight junction and signaling pathways regulating the pluripotency of stem cells in the L. Nu group displayed a trend of enrichment. The L. Nu group was less enriched in the apoptosis pathway than the LPS group (Figure 4I). These findings indicated that the therapeutic effects of NU9056 might be involved in inhibiting apoptosis, promoting tight junctions, and signaling pathways regulating the pluripotency of stem cells.

### NU9056 in LPS-Induced Mice Might Be Associated With SCFA

**Figure 5:**
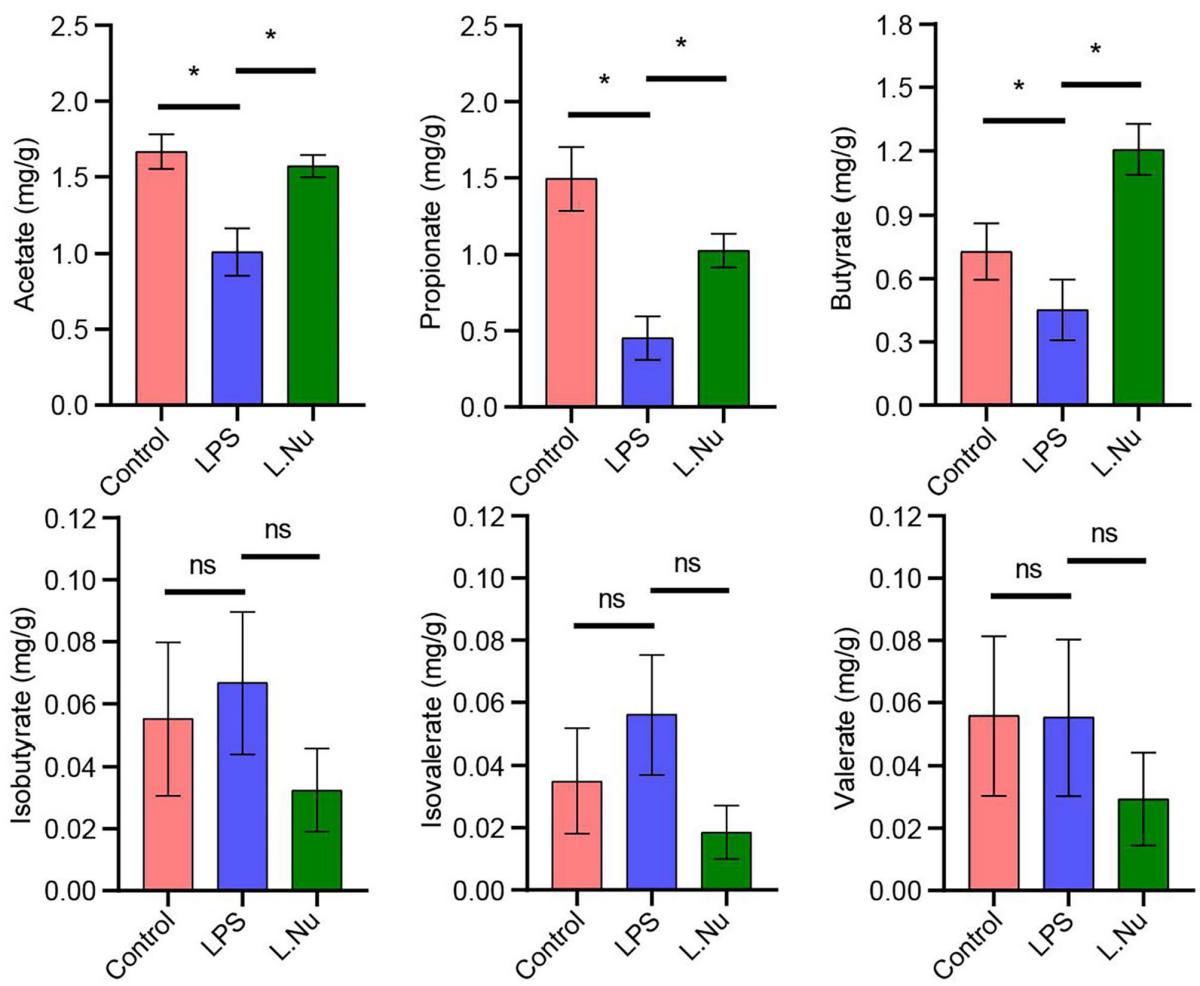
NU9056 in LPS-induced mice might be associated with SCFA. GC-MS determination of the concentration of SCFA consisting of acetate, propionic, butyric, isobutyric, isovalerate, and valerate in the stools of mice. *P < 0.05; ns, not significant.

Changes in the gut microbiota are often closely associated with metabolism. Therefore, we further studied the effects of NU9056 on SCFAs. GC-MS revealed that in contrast with the LPS group, the concentrations of acetate, propionate, and butyric markedly increased, while the overall concentrations of isobutyrate, isovalerate, and valerate were reduced and showed no evident change trend in the L. Nu group (Figure 5). These results suggested that the alleviating effects of NU9056 in LPS-induced mice might be associated with SCFA.

### Gut Microbiota Was Related to SCFA and Inflammatory Factors in LPS-Induced Mice

**Figure 6:**
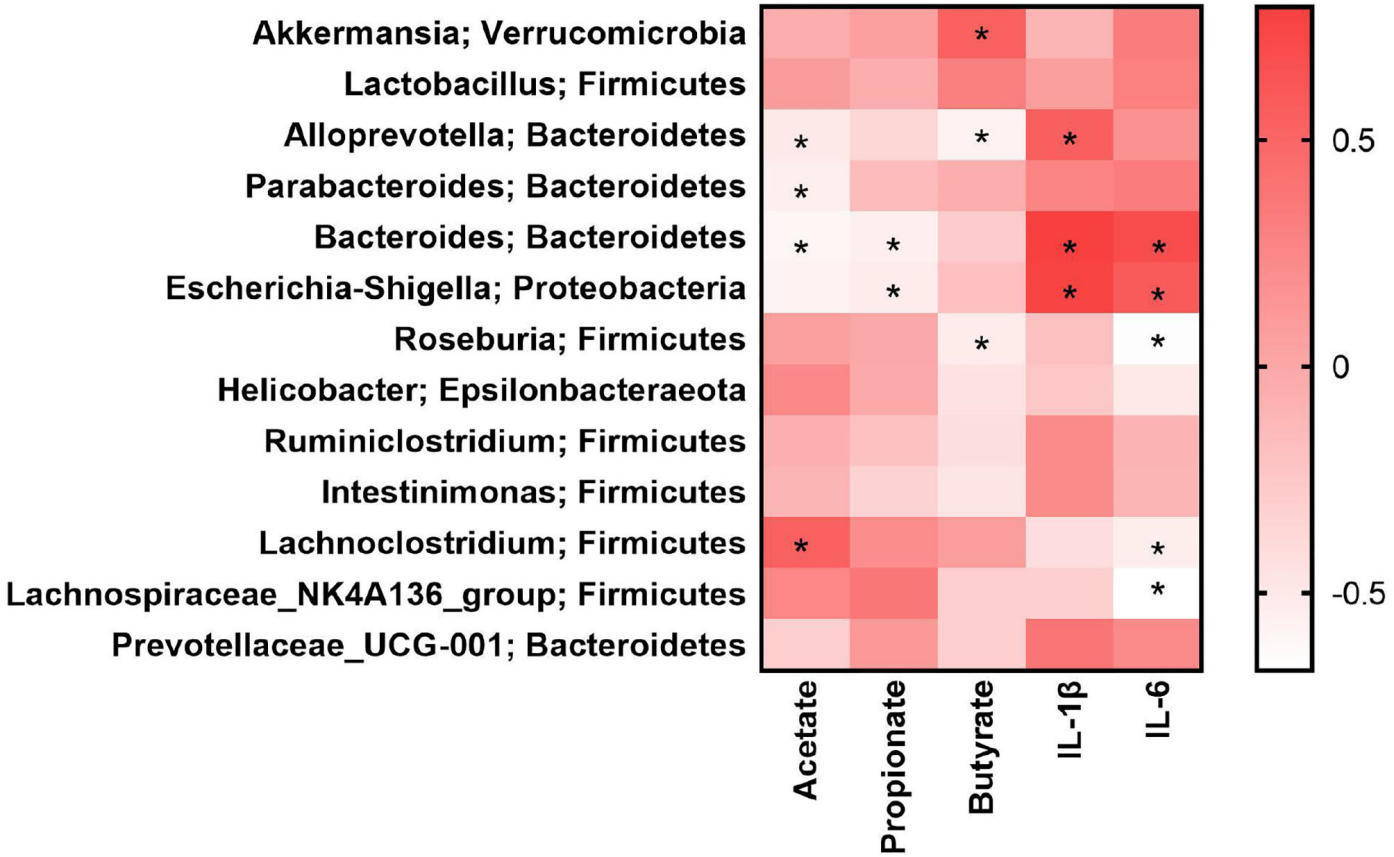
Gut microbiota was related to SCFA and inflammatory factors in LPS-induced mice. *P < 0.05.

The above results indicated that the effects of NU9056 may be related to the gut microbiota, SCFA, and inflammatory factors. To analyze the correlation among them, the Spearman correlation coefficient algorithm was used (Figure 6). The heatmap revealed that the concentration of acetate was negatively correlated with *Alloprevotella* (*r* = −0.51, *P* = 0.036), *Parabacteroides* (*r* = −0.55, *P* = 0.021), and *Bacteroides* (*r* = −0.60, *P* = 0.029), and positively correlated with *Lachnoclostridium* (*r* = 0.54, *P* = 0.041). Propionate was negatively correlated with *Bacteroides* (*r* = −0.55, *P* = 0.036) and *Escherichia-Shigella* (*r* = −0.52, *P* = 0.048). Butyrate was positively correlated with *Akkermansia* (*r* = 0.54, *P* = 0.042), but negatively correlated with *Alloprevotella* (*r* = −0.57, *P* = 0.029) and *Roseburia* (*r* = −0.53, *P* = 0.047). The inflammatory factor IL-1β was significantly positively correlated in *Alloprevotella* (*r* = 0.55, *P* = 0.035), *Bacteroides* (*r* = 0.79, *P* = 0.001), and *Escherichia-Shigella* (*r* = 0.76, *P* = 0.002). The inflammatory factor IL-6 was significantly positively correlated with *Bacteroides* (*r* = 0.70, *P* = 0.005) and *Escherichia-Shigella* (*r* = 0.59, *P* = 0.023). The inflammatory factor IL-6 was significantly negatively associated with *Roseburia* (*r* = −0.66, *P* = 0.009), *Lachnoclostridium* (*r* = −0.54, *P* = 0.041), and *Lachnospiraceae_NK4A136_group* (*r* = −0.67, *P* = 0.008).

### NU9056 Inhibited Apoptosis and Inflammation of Microglia *in vitro*

**Figure 7:**
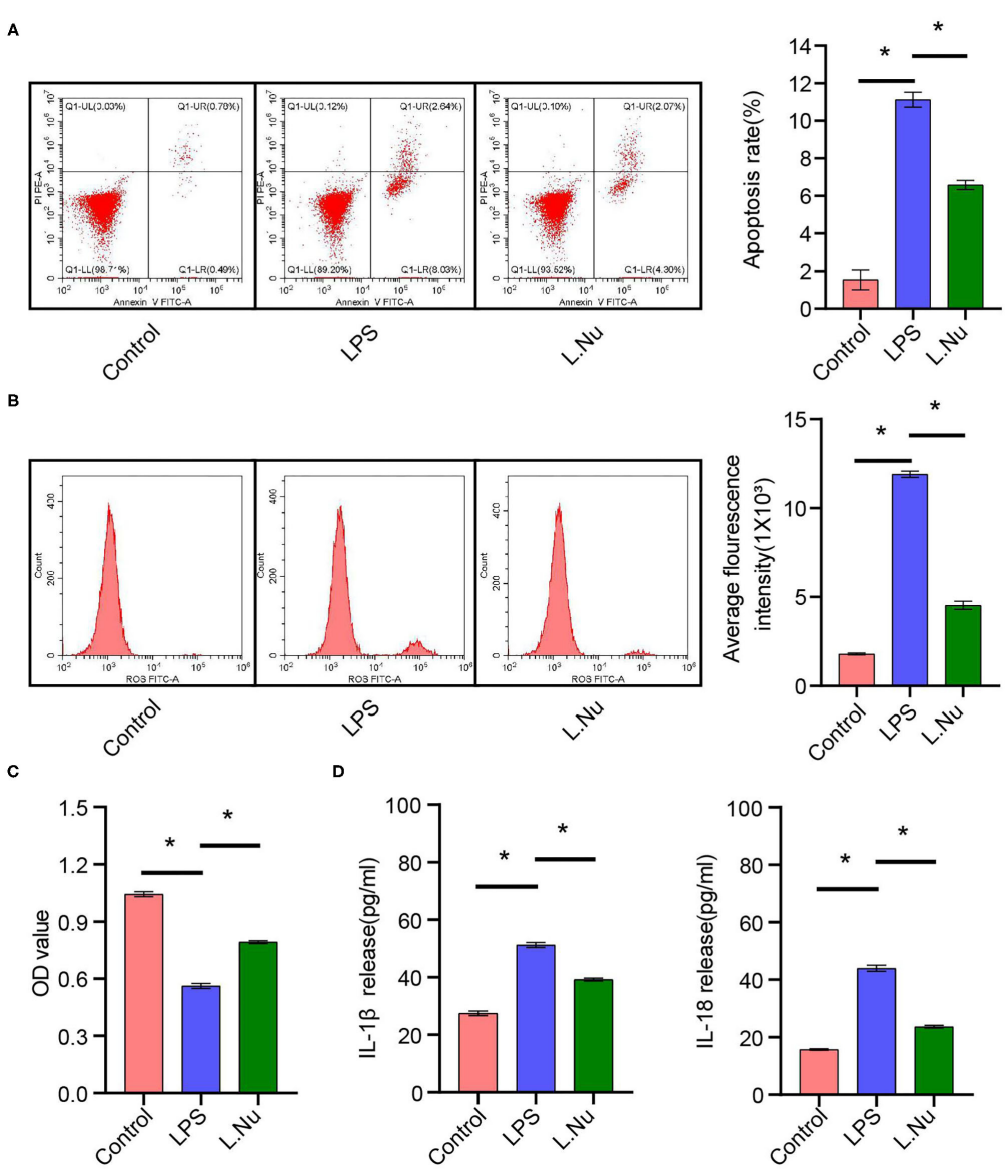
NU9056 inhibited apoptosis and inflammation of microglia *in vitro*. **(A)** Apoptosis rate in each group was detected using flow cytometry. **(B)** The average fluorescence intensity of each group was measured using flow cytometry. **(C)** Cell viability was tested using CCK8 assay. **(D)** The IL-1β and IL-18 release were estimated using ELISA. *P < 0.05.

The above results showed that NU9056 had therapeutic effects on LPS-induced mice. Subsequently, we wanted to verify its protective effects *in vitro* further. We detected the apoptosis rate and ROS levels in each group of cells. Compared to that in the LPS group. The apoptosis rate and average fluorescence intensity of BV2 cells in the L. Nu group were markedly lower (Figures 7A,B). The CCK8 results revealed that the cell viability of the L. Nu group was remarkably higher than that of the LPS group (Figure 7C). We further tested the inflammatory factors. ELISA results revealed that NU9056 suppressed the LPS-induced expression of IL-1β and Il-18 (Figure 7D). The collective results suggested that NU9056 also inhibited apoptosis and inflammation in microglia *in vitro*.

## Discussion

SAE occurs in 70% of patients admitted to the ICU, which might be related to abnormal activation of microglia, brain inflammation, neurotransmitter dysfunction, and other causes (19). In this study, the mitigation effects of NU9056 on LPS-stimulated mouse models of behavioral disorders, brain damage, abnormal activation of microglia, brain inflammation, and BBB function were investigated. NU9056 remarkably improved the survival rate of LPS-stimulated mice, relieved cognitive dysfunction, anxiety, and depression, reduced DCX expression, hindered abnormal activation of microglia, reduced neuroinflammation, protected BBB function, and affected the composition of the gut microbiota. In addition, we validated the results of the LPS-induced inflammation model *in vitro*. The collective findings indicate that NU9056 treatment might have effectively alleviated damage in the LPS model mice by inhibiting the NLRP3 inflammasome to some extent.

The NLRP3 inflammasome is activated in the central nervous system, which can cause many neuroinflammatory diseases (20). The widely accepted view is that NLRP3 binds to ASC after activation and then binds to Casp-1. The active Casp-1 rapidly cleaves pro-IL-1β and pro-IL-18 to mature IL-1β and IL-18, respectively. Subsequently, IL-1β is released outside the cell, causing inflammation (21). IL-6 is another common inflammatory factor. Typically LPS induces an increase in the concentration of IL-6 (22). As an NLRP3 inhibitor, the therapeutic effects of MCC950 on diabetic stroke rats were similar to those of NU9056 in the treatment of LPS-induced mice (23). In this study, we determined the concentrations of IL-1β and IL-6 in the blood of mice in each group *in vivo*. NU9056 could markedly downregulate the concentration of IL-1β without significantly affecting the IL-6 level in the blood. This was consistent with the research of Zhao et al. (15). NU9056 attenuated the release of IL-1β and IL-18 *in vitro*. This might be explained by the fact that NU9056 is a specific inhibitor of KAT5. It may have an anti-inflammatory effect by inhibiting the activation of NLRP3 inflammasomes. However, the downstream pathway of NLRP3 inflammasome may be mainly IL-1β and IL-18 (21). Therefore, NU9056 has no significant effect on IL-6 inflammatory cytokines.

Microglia, BV2 cells, are permanent immune cells in the brain and play an essential role in regulating inflammation in the brain (24). NOD-like receptor protein 3 (NLRP3) was also widely expressed in the cells (25). BV2 cells are often used to study the BBB function, inflammation, NLRP3 pathway, etc. (25, 26). Therefore, BV2 cells have been studied *in vitro*. Due to funding limitations, NU9056 has not been studied on other cells, such as astrocytes and neural cells. We will further explore NU9056 in microglia, astrocytes, neural cells, and other cellular mechanisms related to neuroinflammation in future studies. In addition, abnormal activation of microglia increases ROS levels abnormally and causes apoptosis in brain tissue (20). The present study results also showed that NU9056 could reverse LPS-induced activation of microglia to a certain extent. Activation of TLR4-NF-kB in LPS-induced BV2 cells has been reported in many papers (27, 28). If NU9056 could block the TLR4-NF-kB pathway simultaneously, it would be an essential basis for the possible action of NU9056 on other inflammatory diseases. But due to the funding and time, we did not do that. Future studies will further investigate whether NU9056 can inhibit different inflammatory pathways other than NLRP3, such as TLR4-NF-kB.

BBB damage was involved in the occurrence and development of many neuroinflammatory diseases, including SAE. Therefore, many therapeutic drugs related to neurological diseases have the effect of protecting BBB (29). Our research revealed that NU9056 could alleviate the BBB damage of LPS-induced mice. The result of TEM found that the tight junctions become thicker; additionally, the BBB-related proteins Occludin and Claudin 5 also have an upward trend, suggesting that NU9056 has a relieving effect on BBB. It further illustrated the great potential of NU9056 as a treatment for SAE.

SAEs have been well-established to cause severe cognitive impairment (30). DCX is a classic marker of newborn neurons (31). Furthermore, in depression-like model mice, treatment with ghrelin increased the expression level of DCX (32). Consistent with these findings, we observed that the decrease in the expression level of DCX in the LPS model was reversed by NU9056, suggesting that NU9056 has the potential to protect newborn neurons and further alleviate cognitive impairment.

The cognitive ability of mice has been assessed through OFT, NOR, and other behavioral experiments (33). Liao et al. reported that S100A9 could contribute to the learning and memory impairment of experimental sepsis mice (34). In our study, different behavioral experiments were performed in mice treated with LPS and NU9056. The same animals were used for different behavioral experiments. The reality is that a behavioral experiment such as NOR often takes several hours. After a long period of the behavioral experiment, mice showed unstable mood and abnormal performance. The circadian rhythm also affected the mice's behavior. In addition, there may be some influence between different behavioral experiments. With these factors in mind, in our experiment, the mice were tested by OFT, NOR, EPM, and TST behavioral experiments on days 5, 6, 7, 8, and 9 after LPS modeling. OFT detected the recovery of activity ability of mice in each group. The total movement distance of the three groups of mice within a certain period of time was the same, which meant that their activity ability had returned to a consistent level. This could avoid the deviation of subsequent behavioral tests due to differences in activity ability (35, 36). Then, according to the size of the behavioral stimuli, NOR, EMP, and TST were performed to detect the memory, anxiety, and depression of the mice, respectively. In NOR, EMP, and TST experiments, NU9056 showed the effects of improving memory ability and alleviating anxiety and depression in sepsis mice.

Increasing evidence has shown that inflammation, BBB function, and cognitive dysfunction in SAE mice provide a key link to gut microbiota and metabolism (9). The NLRP3 inflammasome plays a crucial role in coordinating host physiology and shaping the peripheral and central immune and inflammatory responses of central nervous system diseases (37, 38). The “microbiota-gut-brain axis” view has been posited, discussed, and has become a hot research topic (39). Therefore, we speculate that the therapeutic effects of NU9056 may be related to the gut microbiota. *Akkermansia* has been studied recently as a probiotic with great potential. In cancer treatment, *Akkermansia* has great potential to combine with programmed death-1 immunotherapy to enhance its efficacy (40). *Akkermansia* has also been proven to be metabolically beneficial in obese and diabetic mice (41). In the present study, after NU9056 treatment, the abundance of *Akkermansia* in mice was significantly increased, suggesting that NU9056 might attenuate SAE by regulating the gut microbiota. The KEGG prediction results indicated that NU9056 could reduce the accumulation of the apoptosis pathway in the model mice and increase the enrichment of tight junctions and signaling pathways regulating pluripotency of stem cells. This also illustrated the possibility of using NU9056 to alleviate SAEs.

SCFAs have been the main fermentation metabolite of anaerobic bacteria in the gut. SCFAs are the main fermentation metabolites of anaerobic bacteria in the gut. Moreover, they have been considered as potential mediators of the influence of gut microbiota on intestinal immune function (42). The SCFA results revealed that acetate, butyrate, and propionate levels were reduced in patients with encephalitis (43). In the present study, NU9056 notably reversed the LPS-induced decrease in the concentrations of acetate, propionic acid, and butyrate. Due to economic and experimental limitations, its specific effects have not been deeply explored. In future studies, we will investigate the detailed mechanism of NU9056 as a potential SAE treatment. In view of previous studies, *in vivo* and *in vitro* experiments have shown that there is no significant difference between the NU9056 without LPS treatment group and the control group in function and NLRP3 pathway experiments (15, 44, 45). Therefore, in that design, NU9056 was not set without LPS treatment group. Considering the rigor of the experiment, we will set the NU9056 without LPS group in the future study to further study whether NU9056 has any effect on normal animals or cells.

SAE is an acute disease. Patients with SAE may have acute changes in consciousness, which is an important cause of death (19). LPS was used to establish a model that induced acute inflammation in mice, and the inflammatory indicators change significantly during 0–72 h (46). In the acute kidney injury study, inflammatory cytokines such as IL-1β, IL6, and TNF-α were measured in mice 16 h after intraperitoneal injection of LPS (47). Therefore, ELISA was performed at 16 h after modeling in the study. Many studies have shown that BBB markers were detected 24 h after modeling (24). Therefore, the indicators of BBB included Occludin and claudin-5, and the samples were collected 1 day after LPS modeling in the electron microscope experiment. The subsequently affected pathways often take a certain amount of time, so we chose 3 days to study pathways. Due to fund limitations, we did not conduct experiments on the changes of inflammatory indicators and pathway indicators over time. In future studies, we will collect blood and tissues at different time points for further research.

## Conclusion

NU9056 might effectively alleviate the cognitive impairment, emotional disorder, inflammation, and BBB dysfunction of the experimental SAE by inhibiting the NLRP3 inflammasome. In addition, the therapeutic effects of NU9056 on experimental SAE may be related to the gut microbiota and derived metabolites.

## Data Availability Statement

The datasets presented in this study can be found in online repositories. The names of the repository/repositories and accession number(s) can be found at: https://www.ncbi.nlm.nih.gov accession number: PRJNA726029.

## Ethics Statement

The animal study was reviewed and approved by Animal Care and Use Committee of the Third Xiangya Hospital, Central South University.

## Author Contributions

WQ, ZY, and GL had data collection and analysis, and manuscript preparation. LC, QP, and FZ supervised the whole study, data analysis, and manuscript preparation. All authors contributed to the article and approved the submitted version.

## Conflict of Interest

The authors declare that the research was conducted in the absence of any commercial or financial relationships that could be construed as a potential conflict of interest.
