# Supplementary material for: NU9056, a KAT 5 Inhibitor, Treatment Alleviates Brain Dysfunction by Inhibiting NLRP3 Inflammasome Activation, Affecting Gut Microbiota, and Derived Metabolites in LPS-Treated Mice
Source: Front Nutr. 2021 Jul 13;8:701760. doi: 10.3389/fnut.2021.701760 (PMC8313765; doi:10.3389/fnut.2021.701760)
Supplement: Supplementary file 1 [file Image_1.pdf]

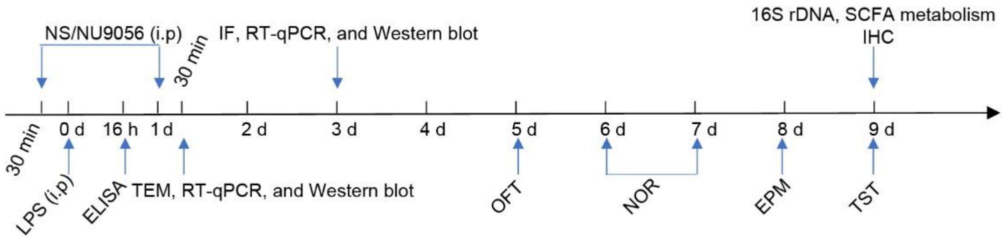

Supplementary Figure 1. Mice treatment design. Twelve-week-old mice were intraperitoneally injected with NU9056 30 minutes before and 1 day after LPS intraperitoneal injection. OFT, NOR, EPM, and TST behavioral tests were performed 5, 6, 7, 8, and 9 days after LPS treatment. Animal serum, tissue, or stool samples were collected 16 h, 1, 3, and 9 days after intraperitoneal injection of LPS.
